# Morphometric study of *Kalophrynus palmatissimus* at two forest reserves in Malaysia

**DOI:** 10.1002/ece3.7721

**Published:** 2021-07-27

**Authors:** Muhammad Faris Abdul Aziz, Marina Mohd. Top @ Mohd. Tah, Shamarina Shohaimi, Nurul Izza Ab Ghani, Christine Fletcher

**Affiliations:** ^1^ Department of Biology Faculty of Science Universiti Putra Malaysia Serdang Malaysia; ^2^ Centre of Foundation Studies for Agricultural Science Universiti Putra Malaysia Serdang Malaysia; ^3^ Pasoh FRIM Research Station Forest Research Institute Malaysia (FRIM) Kepong Malaysia

**Keywords:** climatic factors, forest reserve, *Kalophrynus palmatissimus*, morphometrics, transect lines

## Abstract

A research study on morphometrics of *Kalophrynus palmatissimus* (commonly known as Lowland Grainy Frog) at Ayer Hitam Forest Reserve (AHFR), Selangor and Pasoh Forest Reserve (PFR), Negeri Sembilan was carried out from 12 November 2016 to 13 September 2017. The study was to examine data on the morphometric traits of *K. palmatissimus* at the two forest reserves. 15 morphometric traits of *K. palmatissimus* that were taken by using vernier calipers. Frog surveys were done by using 15 and 18 nocturnal 400 m transect lines with an interval distance of 20 m at AHFR and PFR, respectively. The GPS coordinates for all frog samples were recorded to ensure the precise geographic location. In addition, five climatic data were recorded. The results showed that most morphometric traits in AHFR (*n* = 34) and PFR (*n* = 31) were positively correlated with each other. On the other hand, climatic factor, which was soil pH, had a significant positive influence on most of the morphometric traits (*p* < .01), except for tympanum diameter and upper eyelid width (*p* ≥ .05). Meanwhile, the temperature had a significantly negative influence on all morphometric traits (*p* < .01). General linear model (GLM) analysis showed that snout‐vent length (SVL) influenced most morphometric traits (*F* ≤ 80.86, *p* < .01), except for hand length (HAL: *F* = 0.299, *p* > .05). Later, it was found that the snout‐vent length of *K. palmatissimus* at AHFR was slightly larger than at PFR (AHFR: *μ* = 37.00 mm, *SE* = 1.16 *c.f*. PFR: *μ* = 30.29 mm, *SE* = 1.07). It showed that there were variations in morphometric traits of *K. palmatissimus* at AHFR and PFR. From PCA analysis, morphometric traits are grouped into two components for AHFR and PFR, respectively. In AHFR, head length, eye diameter, head width, internarial distance, interorbital distance, forearm length, tibia length, foot length, and thigh length were strongly correlated, while snout length and eye‐nostril distance were strongly correlated. In PFR, eye diameter, head width, internarial distance, interorbital distance, foot length, and thigh length were strongly correlated, though snout length and eye‐nostril distance were strongly correlated, hence, suggested that all morphometric traits grow simultaneously in *K. palmatissimus* with eye‐nostril distance (EN), and snout length (SL) growing almost simultaneously at AHFR (*r* = .91) and PFR (*r* = .97). There is still a lack of available information regarding the distribution and morphometric studies of *K. palmatissimus* in Malaysia, especially at AHFR and PFR. This study showed 15 different morphometric traits of *K. palmatisssimus* between AHFR and PFR, with *K. palmatissimus* at AHFR were found to be slightly larger than at PFR.

## Graphical abstract

A research study on morphometrics of *Kalophrynus palmatissimus* (commonly known as Lowland Grainy Frog) at Ayer Hitam Forest Reserve (AHFR), Selangor and Pasoh Forest Reserve (PFR), Negeri Sembilan was carried out from 12 November 2016 to 13 September 2017. The study was conducted to examine data on the morphometric traits of *K. palmatissimus* at the two forest reserves. The data collections showed the 15 different morphometric traits of *K. palmatissimus* between AHFR and PFR with *K. palmatissimus* at AHFR were slightly larger than at PFR.

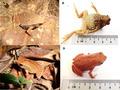

## 1 BACKGROUND

The genus *Kalophrynus* is reported to consist of 25 nominal species with the greatest diversity in Borneo (Zug, 2015). Six species previously reported in Peninsular Malaysia are *Kalophrynus limbooliati*, *Kalophrynus palmatissimus*, *Kalophrynus pleurostigma, Kalophrynus robinsoni, Kalophrynus tiomanensis, and Kalophrynus yongi* (Zug, 2015). These species are usually found at relatively low elevations, and the highest known record was 1,006 m above sea level (a.s.l) for *K*. *robinsoni* (Dring, 1979). Manthey and Grossmann (1997) reported that it usually inhabit or habituate undisturbed lowland rainforests and bamboo stands. Meanwhile, their reproduction sites include water‐filled bamboo stumps and other cavities (IUCN, 2016).

From those six reported species in Malaysia, according to IUCN Red List 2016, *Kalophrynus palmatissimus* is listed as endangered species, and it is protected under the Wildlife Conservation Act 2010. This species is endemic to Malaysia and reported to exist only at Pasoh Forest Reserve, Gombak Forest Reserve, FRIM, Templer's Park in Selangor (IUCN, 2016), and Ayer Hitam Forest Reserve, Puchong (Muhammad Faris et al., 2016). *Kalophrynus palmatissimus* is a species of frog from the Microhylidae family. However, its phylogenetic relations with other Microhylidae family are unclear (Frost et al., 2006), and now it is considered to represent a distinct subfamily, *Kalophryninae* (Frost, 2009). Phylogenetic relationships are assessed either by using a classical approach or molecular approach, or a combination of both approaches. The classical approach refers to morphological characteristics and morphometric traits. Morphometric traits of herpetological organisms are necessary for species delineation, phylogenetic analyses, and understanding of evolutionary changes in an organism's physical characteristics, but yet there are few consistencies in physical measurements and descriptions across, or even within taxonomy (Dubois, 2010). Arbour and Brown (2014) assigned that the accurate measurement of amphibian morphometrics is essential for taxonomy, studies of growth and development, and studies of fluctuating asymmetry. According to Singleton et al. (2011), morphometric or morphometry is a quantitative analysis form, which is a concept that divided into size and shape. In frogs, 12–16 morphometric traits are used to distinguish species (Chan et al., 2011; Kiew, 1984; Watters et al., 2016).

In this study, *K. palmatissimus* from two populations: Ayer Hitam Forest Reserve (AHFR), Selangor and Pasoh Forest Reserve (PFR), Negeri Sembilan, studied for 15 morphometric traits. According to Matsui (1984), there were 15 body measurements of *K. palmatissimus* were taken in millimeters (mm), since there is a lack of study on the detailed information concerning the morphometric traits differentiation of these species in Peninsular Malaysia especially at AHFR and PFR. Though several studies conducted, the studies only measured 13 morphometric traits (Kiew, 1984) and two morphometric traits (Muhammad Faris, 2016) for species from Ayer Hitam Forest Reserve (AHFR), Selangor with fewer individuals (*n* = two). Furthermore, previous studies only focused on morphometric traits of *K. palmatissimus* with different populations separately (Kiew, 1984). In the aspect of environmental parameters, air temperature (°C), light intensity (lx), wind (m/s), soil pH, and relative humidity (%RH) of two forest reserves were determined and recorded. Therefore, the objectives of this study are to determine the morphometric traits differences of *K. palmatissimus* at the two previously studied forest reserves (AHFR and PFR) and the potential correlations among morphometric traits and climatic factors.

## 2 MATERIALS AND METHODS

### 2.1 Study sites

**FIGURE 1:**
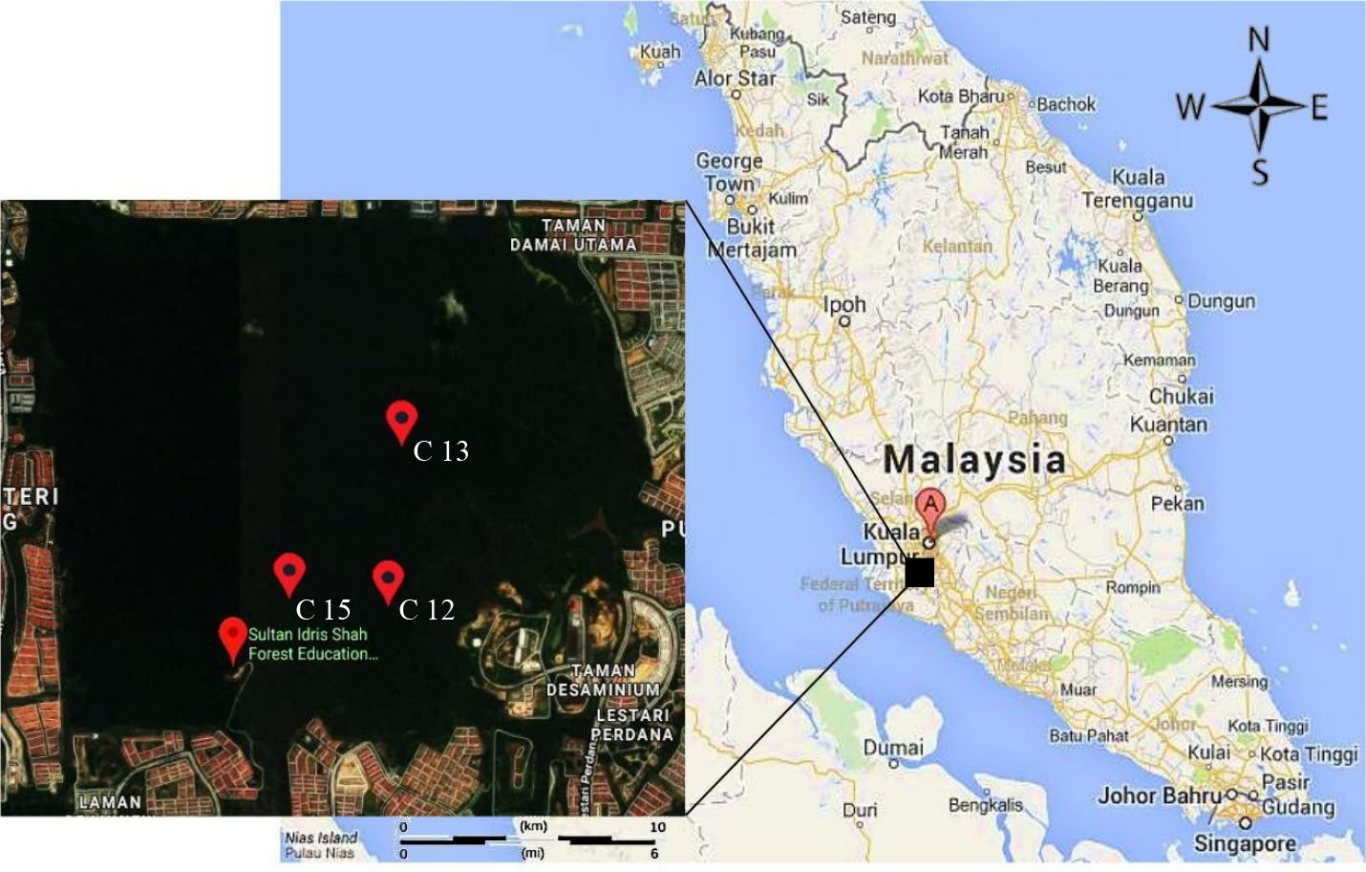
Location of compartments 12, 13, and 15, Ayer Hitam Forest Reserve, Puchong, Selangor (AHFR)

**FIGURE 2:**
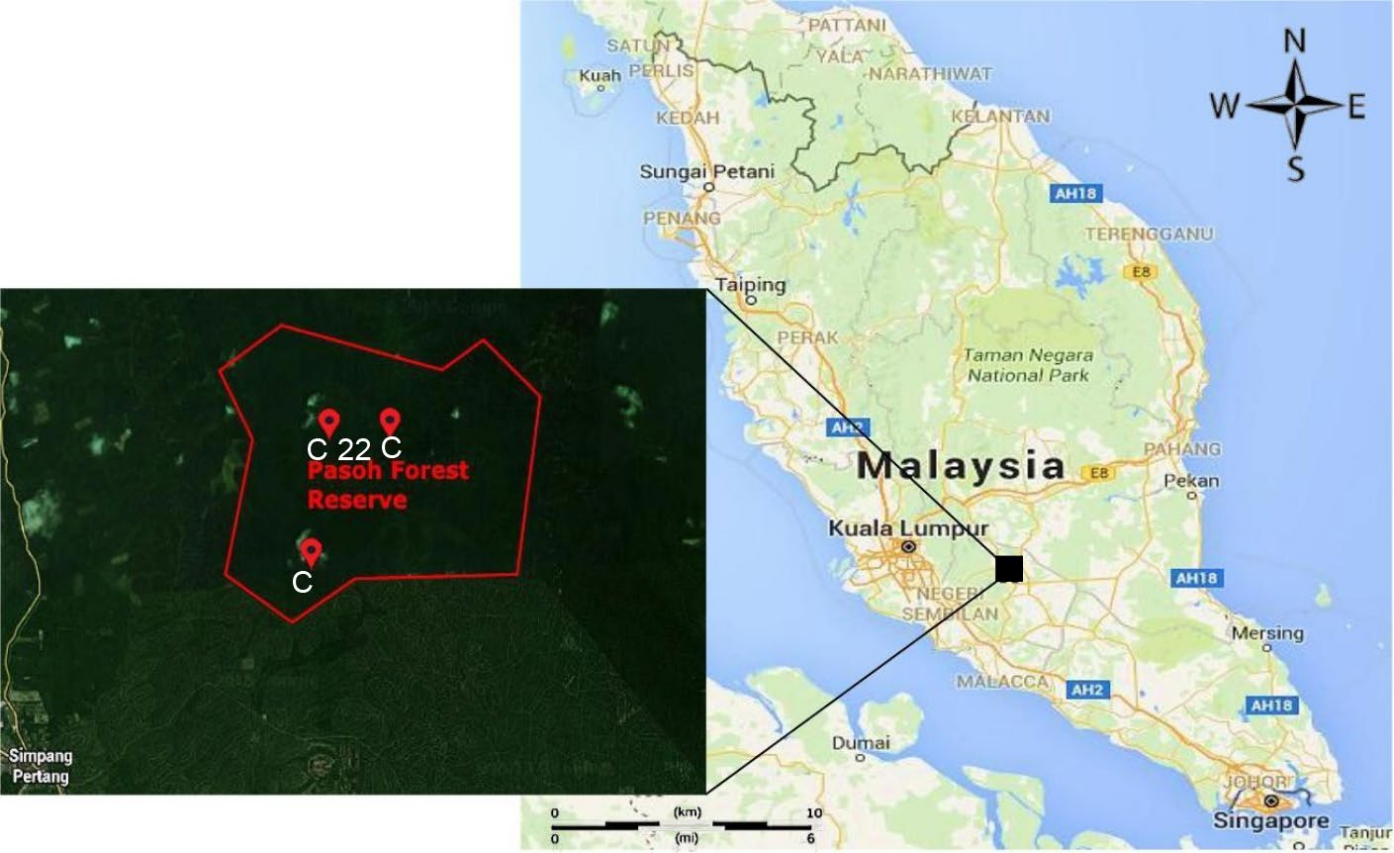
Location of compartment 21, 22 and 32, Pasoh Forest Reserve, Negeri Sembilan (PFR)

The project conducted at two study sites, which were Ayer Hitam Forest Reserve (AHFR), Selangor and Pasoh Forest Reserve (PFR), Negeri Sembilan from 12 November 2016 to 13 September 2017. The Ayer Hitam Forest Reserve (AHFR) is 15 to 233 m above sea level with its highest peak at Bukit Permatang Kumbang. The Rasau River at the south and Bohol River at the north are the primary sources of irrigation in this forest (Paiman & Amat, 2007; Shamsudin et al., 2015). The three studied compartments of AHFR were Compartment 12, Compartment 13, and Compartment 15 (Figure 1). Meanwhile, the core area of Pasoh Forest Reserve (PFR), which is approximately 600 ha, is still covered with old‐growth forest. However, most of the surrounding areas of this forest logged in the past. Therefore, this forest is an example of a regenerating lowland forest. The PFR has an area of approximately 140 km^2^, covered with lowland dipterocarp forest and hill dipterocarp forest at its north‐eastern boundary. The three studied PFR compartments were Compartment 21, Compartment 22, and Compartment 32 (Figure 2).

### 2.2 Sampling of *Kalophrynus palmatissimus*

During this study, a visual encounter survey (VES) was utilized as the sampling method. VES helps to maximize the observation of frogs in each survey so that the optimum number of frog species per survey can be achieved (Crump & Scott, 1994). Surveys on *K. palmatissimus* were conducted at two different study areas which were located at walking trails and river banks based on the accessibility of these areas. Fifteen and eighteen 400 m nocturnal transect lines with an interval distance of 20 m were established for frog surveys in AHFR and PFR, respectively. Gillespie (1997) recommended that 400 m to 500 m transect lines along riparian habitat were the preferred distance for a VES to study threatened frog species.

The visual encounter survey procedure consisted of an active search for frogs in a randomized walk at a steady pace from November 2016 to September 2017. At AHFR, the frog surveys were conducted for eight nights per month within a five months of the sampling period. In PFR, the surveys were conducted for 12 nights per month within a six‐month sampling period. The duration for each survey was between three and four hours, which started at 2030 and finished at 0000, involving two to three personnel to search for the presence of *K. palmatissimus*.

The coordinates of captured *K. palmatissimus* were also taken by using a GPS device (Garmin GPSMAP 64S Handheld) to ensure precise geographic location records. Prior to the survey, climatic factors, such as macroclimates and microclimates data (i.e., temperature, wind, light intensity, humidity, and soil pH) were recorded by using meteorological apparatus (Extech Meter for Humidity, Temperature, Airflow and Light Level 45179) and pH soil device (pH soil and moisture tester Takemura DM‐15 for soil pH).

Captured frogs were stored in plastic containers (12 cm × 7 cm) and brought back to the on‐site laboratories. The captured frogs were weighed by using a weighing scale (PESOLA Weighing Scale), and photographs of the frogs at every viewpoint were taken for species identification. Species identification was made by referring to the online database, namely “Amphibia.my: Amphibians & Reptiles of Peninsular Malaysia,” and reference books entitled “Amphibians and Reptiles of the Seribuat Archipelago of Peninsular Malaysia” by Grismer (2011) and “The Amphibian Fauna of Peninsular Malaysia” by Berry (1975).

### 2.3 Morphometric traits measurement

Fifteen morphometric traits of *K. palmatissimus* were taken in millimeter (mm) units of the length, according to Matsui (1984) by using vernier calipers. The measurements were as follows: (1) snout‐vent length (SVL), (2) head length (HL), (3) snout length (SL), (4) eye‐nostril distance (EN), (5) eye diameter (ED), (6) Tympanum diameter (TD), (7) head width (HW), (8) internarial distance (IND), (9) interorbital distance (IOD), (10) upper eyelid width (UEW), (11) hand length (HAL), (12) forelimb length (FLL), (13) tibia length (TL), (14) foot length (FL), and (15) thigh length (THL). The samples were individually examined and measured at AHFR and PFR on‐site Laboratory. After taken all of the measurements, blue nail polish was used as a dye to mark the tibia of each *K. palmatissimus* before released it back at the captured area. It was done to avoid any recapture of *K. palmatissimus* individual. The frogs were ethically handled during the study (UPM/IACUC/AUP‐R007/2018).

### 2.4 Data analysis

To evaluate sampling effort and relate the morphometric traits of *K. palmatissimus* between two forest reserves, individuals of this species were assessed based on the presence or absence at the study areas (walking trails and river banks). All statistical analyses were done separately for individuals from AHFR and PFR. The independent samples *t* test was performed to evaluate sexual dimorphism since it is known that *K. palmatissimus* is a sexually dimorphic animal (Kiew, 1984). According to Tolosa et al. (2015), sexual dimorphism in anuran species are body size (female is larger than male), tympanum size (male has tympanum size larger than eye while the female has tympanum size equal or smaller than the eye), and the throat coloration, which is a dark‐spotted coloration in males, while light color in females. Pearson's correlation coefficient test was used to address the association between 15 morphometric traits to evaluate the significant correlation of morphometric traits within them. Meanwhile, the association between climatic factors, which are microclimates data and morphometric traits, were calculated by using Spearman's rank correlation coefficient to assess the association of those factors on the morphometric traits. General linear mix model (GLM) was used to evaluate which factors: snout‐vent length or soil pH A or soil pH B or habitat or collective factors of sex and SVL (sex × SVL) or soil pH B and SVL (soil pH B × SVL), had influenced the morphometric traits of *K. palmatissimus*. Principle component analysis (PCA) was conducted to further understand the growth pattern of all morphometric traits. All statistical analyses performed by using IBM SPSS Statistics version 22.0 (SPSS, Inc, Chicago, IL, USA) (IBM Corp, 2013).

## 3 RESULTS

**FIGURE 3:**
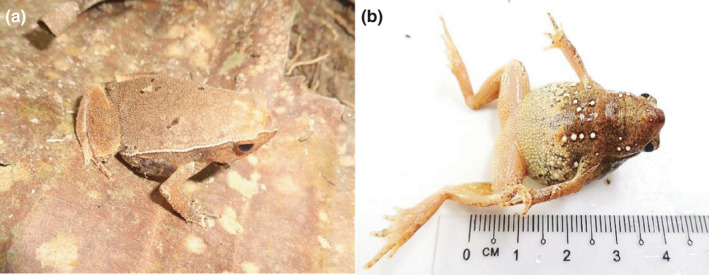
Images of *Kalophrynus palmatissimus* at Ayer Hitam Forest Reserve, Puchong, Selangor (AHFR). (a) Lateral and (b) ventral views

**FIGURE 4:**
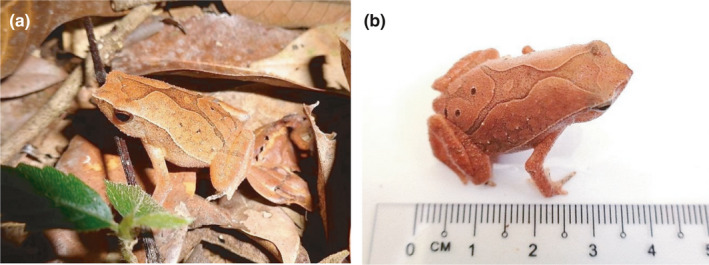
Images of *Kalophrynus palmatissimus* at Pasoh Forest Reserve, Negeri Sembilan (PFR). (a) Lateral and (b) dorsal views

A total of 65 samples of *K. palmatissimus* were captured and recorded, including 34 samples (males: 20, females: 14) from Ayer Hitam Forest Reserve, Selangor (AHFR; Figure 3) and 31 samples (males: 20, females: 11) from Pasoh Forest Reserve, Negeri Sembilan (PFR Figure 4). The comparisons of 15 morphometric traits between *K. palmatissimus* at AHFR and PFR has shown in Table 1. The descriptive statistics showed that morphometric traits of *K. palmatissimus* at AHFR were slightly larger than the morphometric traits of *K. palmatissimus* at PFR. The Ayer Hitam Forest Reserve (AHFR) and Pasoh Forest Reserve (PFR) samples also showed size dimorphism with females as the larger sex. At AHFR, the females had a lengthier head length (HL), eye diameter (ED), and head width (HW) than males (Table 2). At PFR, the females had head length (HL), eye diameter (ED), head width (HW), snout‐vent length (SVL), internarial distance (IND), and thigh length (THL) longer than the males (Table 3).

**TABLE 1:** Descriptive statistics of 15 morphometric traits of *K. palmatissimus* at Ayer Hitam Forest Reserve, Puchong, Selangor (AHFR) and Pasoh Forest Reserve, Negeri Sembilan (PFR), respectively

Morphometric traits	Study site	Number of captured site (*n*)	Range (mm)	Mean ± standard deviation	Standard error
HL	AHFR	34	4.00–14.00	8.09 ± 1.88	0.32
	PFR	31	5.00–9.50	6.84 ± 1.14	0.21
HW	AHFR	34	4.00–10.00	8.04 ± 1.47	0.25
	PFR	31	4.50–9.50	6.92 ± 1.27	0.23
SVL	AHFR	34	18.00–47.00	37.00 ± 6.78	1.16
	PFR	31	20.00–44.00	30.29 ± 5.96	1.07
ED	AHFR	34	2.00–4.50	3.43 ± 0.60	0.10
	PFR	31	2.00–4.50	2.82 ± 0.69	0.12
EN	AHFR	34	1.50–8.50	3.50 ± 1.28	0.22
	PFR	31	1.50–3.50	2.50 ± 0.52	0.09
IND	AHFR	34	1.50–4.00	2.93 ± 0.63	0.11
	PFR	31	1.50–3.00	2.07 ± 0.42	0.08
IOD	AHFR	34	2.00–7.00	4.84 ± 1.03	0.18
	PFR	31	2.50–5.00	3.69 ± 0.65	0.12
UEW	AHFR	34	1.00–3.50	2.50 ± 0.59	0.10
	PFR	31	1.50–3.00	2.23 ± 0.56	0.10
SL	AHFR	34	2.50–9.50	4.56 ± 1.54	0.26
	PFR	31	2.50–4.50	3.50 ± 0.52	0.09
TD	AHFR	34	2.00–4.50	2.99 ± 0.62	0.11
	PFR	31	1.00–3.50	2.42 ± 0.62	0.11
FLL	AHFR	34	4.50–13.00	10.34 ± 2.04	0.35
	PFR	31	5.00–12.00	8.15 ± 1.81	0.33
HAL	AHFR	34	3.50–12.00	8.37 ± 1.62	0.28
	PFR	31	5.00–9.00	6.76 ± 1.03	0.19
TL	AHFR	34	8.00–18.00	15.12 ± 2.26	0.39
	PFR	31	9.00–17.00	12.11 ± 2.25	0.40
THL	AHFR	34	9.00–21.00	16.28 ± 2.69	0.46
	PFR	31	9.00–20.00	13.82 ± 2.68	0.48
FL	AHFR	34	6.50–21.00	13.13 ± 2.79	0.48
	PFR	31	7.00–14.00	9.58 ± 1.96	0.35

Abbreviations: ED, eye diameter; EN, eye‐nostril distance; FL, foot length; FLL, forearm length; HAL, hand length; HL, head length; HW, head width; IND, internarial distance; IOD, interorbital distance; SL, snout length; SVL, snout‐vent length; TD, tympanum diameter; THL, thigh length; TL, tibia length; UEW, upper eyelid width.

**TABLE 2:** Spearman's correlation between microclimates and morphometric traits at Ayer Hitam Forest Reserve (AHFR), Selangor and Pasoh Forest Reserve (PFR), Negeri Sembilan

Spearman's rho	SVL	HL	SL	EN	ED	TD	HW	IND	IOD	UEW	HAL	FLL	TL	FL	THL
Soil pH	0.435**	0.426**	0.382**	0.313*	0.460**	0.228	0.346**	0.274*	0.385**	0.241	0.324**	0.412**	0.439**	0.398**	0.381**
Humidity	−0.054	−0.026	−0.123	−0.224	0.117	−0.097	−0.132	−0.146	−0.150	0.131	−0.132	−0.090	−0.029	−0.093	0.199
Temperature	−0.557**	−0.504**	−0.338**	−0.366**	−0.529**	−0.377**	−0.615**	−0.663**	−0.570**	−0.285*	−0.534**	−0.557**	−0.606**	−0.583**	−0.516**

Abbreviations: ED, eye diameter; EN, eye‐nostril distance; FL, foot length; FLL, forearm length; HAL, hand length; HL, head length; HW, head width; IND, internarial distance; IOD, interorbital distance; SL, snout length; SVL, snout‐vent length; TD, tympanum diameter; THL, thigh length; TL, tibia length; UEW, upper eyelid width.

**Correlation is significant at the 0.01 level (2‐tailed).

*Correlation is significant at the 0.05 level (2‐tailed).

**TABLE 3:** The independent sample *t* test related to morphometric traits and sex of *Kalophrynus palmatissimus* in Ayer Hitam Forest Reserve (AHFR), Selangor (*n* of males = 20), (*n* of females = 14)

Variables	Sex	Mean (mm)	*SD*	*t*	*p*‐value
Head width	Male	0.76	0.16	−2.088	.045
	Female	0.86	0.09		
Snout‐vent length	Male	3.53	0.79	−1.807	.080
	Female	3.94	0.37		
Tibia length	Male	1.46	0.27	−1.634	.112
	Female	1.59	0.12		
Interorbital distance	Male	0.46	0.11	−1.471	.151
	Female	0.51	0.09		
Head length	Male	0.75	0.18	−2.430	.021
	Female	0.90	0.16		
Eye diameter	Male	0.33	0.06	−2.142	.040
	Female	0.37	0.05		
Internarial distance	Male	0.28	0.07	−1.129	.267
	Female	0.31	0.04		
Eye‐nostril distance	Male	0.33	0.12	−0.952	.348
	Female	0.38	0.15		
Foot length	Male	1.25	0.27	−1.551	.131
	Female	1.40	0.27		
Tympanum diameter	Male	0.29	0.06	−0.955	.347
	Female	0.31	0.06		
Thigh length	Male	1.56	0.30	−1.974	.057
	Female	1.73	0.18		
Snout length	Male	0.45	0.16	−0.486	.630
	Female	0.47	0.15		
Hand length	Male	0.81	0.17	−1.160	.254
	Female	0.88	0.15		
Forearm length	Male	0.98	0.23	−1.813	.079
	Female	1.11	0.14		
Upper eyelid width	Male	0.24	0.06	−1.504	.142
	Female	0.27	0.05		

Most of the 15 morphometric traits of *K. palmatissimus* at Ayer Hitam Forest Reserve and Pasoh Forest Reserve were found to have an individually significant correlation (*p* < .05) with each other (Table 4). However, snout length and eye diameter, eye‐nostril distance and eye diameter, eye diameter and tympanum diameter, tympanum diameter and upper eyelid width, internarial distance and eye‐nostril distance, hand length and snout length, and hand length and eye diameter pairs of morphometric traits, were not significantly correlated (*p* > .05; Table 4). Since SVL found significantly correlated with the other 14 morphometric traits (*p* < .05; Table 5), SVL was included as a covariate in the later GLM analysis as a confounding factor. On the other hand, two out of three climatic factors (i.e., soil pH and temperature) showed a significant influence on morphometric traits (Table 6). Soil pH had a significant positive influence on most morphometric traits (*p* < .01), except for tympanum diameter and upper eyelid width (*p* ≥ .05). Meanwhile, temperature had a significant negative influence on all morphometric traits (*p* < .01). The SVL of *K. palmatissimus* found to be significantly associated with all morphometric traits, except for the species hand length (Table 5). Two climatic factors which had significant relation with a few morphometric traits of *K. palmatissimus* at the two forest reserves (*p* < .05, Table 5). The soil pH A (a macroclimate factor) had significantly influenced the head length and foot length (Table 5); however, the soil pH B (a microclimate factor) had significantly influenced snout length, eye‐nostril distance, and upper eyelid width (Table 5). The habitats (AHFR and PFR) had significantly influenced the eye diameter, internarial distance, and thigh length (Table 5). Meanwhile, the head width and tibia length of *K. palmatissimus* were significantly influenced by the interaction between sex and SVL (Table 5). The snout length, eye‐nostril distance, and upper eyelid width of *K. palmatissimus* were significantly influenced by the interaction between soil pH B and SVL (Table 5).

**TABLE 4:** Pearson's correlation between 15 morphometric traits at two forest reserves (Above: Pasoh Forest Reserve (PFR), Negeri Sembilan, Below: Ayer Hitam Forest Reserve (AHFR), Selangor)

Pearson's *r*	SVL	HL	SL	EN	ED	TD	HW	IND	IOD	UEW	HAL	FLL	TL	FL	THL
SVL		0.888**	0.655**	0.655**	0.787**	0.561**	0.874**	0.745**	0.788**	0.678**	0.762**	0.923**	0.938**	0.900**	0.713**
HL	0.753**		0.607**	0.607**	0.639**	0.439*	0.791**	0.694**	0.678**	0.826**	0.694**	0.852**	0.867**	0.860**	0.774**
SL	0.598**	0.491**		1.000**	0.234	0.364*	0.459**	0.381*	0.493**	0.605**	0.485**	0.748**	0.639**	0.543**	0.398*
EN	0.566**	0.525**	0.799**		0.234	0.364*	0.459**	0.381*	0.493**	0.605**	0.485**	0.748**	0.639**	0.543**	0.398*
ED	0.776**	0.597**	0.379*	0.343*		0.355	0.775**	0.640**	0.707**	0.495**	0.465**	0.628**	0.782**	0.787**	0.542**
TD	0.745**	0.512**	0.578**	0.457**	0.582**		0.565**	0.370*	0.553**	0.198	0.541**	0.463**	0.401*	0.402*	0.387*
HW	0.900**	0.757**	0.350*	0.440**	0.729**	0.607**		0.695**	0.704**	0.614**	0.661**	0.801**	0.789**	0.764**	0.728**
IND	0.792**	0.619**	0.372*	0.329	0.503**	0.578**	0.758**		0.766**	0.639**	0.629**	0.791**	0.729**	0.576**	0.415*
IOD	0.857**	0.719**	0.499**	0.496**	0.614**	0.720**	0.818**	0.696**		0.468**	0.615**	0.720**	0.756**	0.695**	0.472**
UEW	0.750**	0.620**	0.625**	0.502**	0.594**	0.599**	0.664**	0.632**	0.687**		0.501**	0.762**	0.714**	0.650**	0.533**
HAL	0.614**	0.611**	0.292	0.374*	0.261	0.466**	0.651**	0.609**	0.630**	0.429*		0.768**	0.721**	0.668**	0.5632*1*
FLL	0.944**	0.815**	0.550**	0.560**	0.739**	0.673**	0.880**	0.769**	0.887**	0.723**	0.591**		0.919**	0.784**	0.634 **
TL	0.955**	0.708**	0.560**	0.523**	0.715**	0.691**	0.894**	0.831**	0.855**	0.771**	0.636**	0.931**		0.928**	0.6824
FL	0.799**	0.637**	0.480**	0.444**	0.564**	0.627**	0.711**	0.762**	0.754**	0.608**	0.543**	0.789**	0.822**		0.749 **
THL	0.767**	0.579**	0.479**	0.437**	0.699**	0.565**	0.646**	0.515**	0.582**	0.664**	0.391*	0.737**	0.797**	0.519**	

Abbreviations: ED, eye diameter; EN, eye‐nostril distance; FL, foot length; FLL, forearm length; HAL, hand length; HL, head length; HW, head width; IND, internarial distance; IOD, interorbital distance; SL, snout length; SVL, snout‐vent length; TD, tympanum diameter; THL, thigh length; TL, tibia length; UEW, upper eyelid width.

**Correlation is significant at the 0.01 level (2‐tailed).

*Correlation is significant at the 0.05 level (2‐tailed).

**TABLE 5:** General linear model of 14 morphometric traits with studied factors at Ayer Hitam Forest Reserve (AHFR), Selangor and Pasoh Forest Reserve (PFR), Negeri Sembilan

Morphometric traits/Studied factors	HL	SL	EN	ED	TD	HW	IND	IOD	UEW	HAL	FLL	TL	FL	THL
S.V.L	15.548**	31.586**	36.661**	8.723*	13.706**	16.156**	5.418*	21.641**	17.281**	0.299	32.305**	49.440**	80.858**	9.269*
Soil pH A	5.670**	1.778	2.509	0.771	1.006	0.486	1.299	2.049	1.969	1.463	0.864	0.552	4.823*	2.779
Soil pH B	1.283	3.495*	8.441**	0.671	1.391	0.862	1.746	1.260	4.075*	1.685	0.709	1.054	2.740	0.612
Habitat	1.001	0.064	0.202	6.399*	0.125	0.603	5.917*	4.704	0.939	1.129	0.315	0.016	0.105	7.583*
Sex × S.V.L	0.213	3.576	2.262	1.314	2.087	5.941*	0.353	1.116	0.989	3.592	0.248	5.008*	0.275	1.101
Soil pH B × S.V.L	1.301	3.537*	9.082**	0.790	1.310	0.830	1.950	1.258	4.371*	1.736	0.747	1.179	2.521	0.646

Abbreviations: ED, eye diameter; EN, eye‐nostril distance; FL, foot length; FLL, forearm length; HAL, hand length; HL, head length; HW, head width; IND, internarial distance; IOD, interorbital distance; SL, snout length; SVL, snout‐vent length; TD, tympanum diameter; THL, thigh length; TL, tibia length; UEW, upper eyelid width.

**Correlation is significant at the 0.01 level (2‐tailed).

*Correlation is significant at the 0.05 level (2‐tailed).

**TABLE 6:** Rotated Component matrix of morphometric parameters in Pasoh Forest Reserve (PFR), Negeri Sembilan

Morphometric traits	Component	
	1	2
Snout length	–	0.97
Eye‐nostril distance	–	0.97
Eye diameter	0.92	–
Head width	0.88	–
Internarial distance	0.78	–
Interorbital distance	0.80	–
Foot length	0.83	–
Thigh length	0.70	–

Note

–, Not applicable.

**FIGURE 5:**
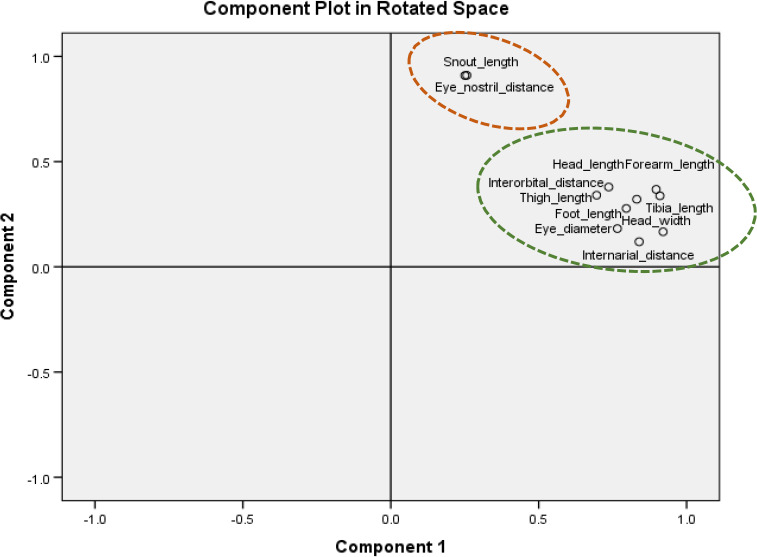
Component matrix of morphometric parameters at Ayer Hitam Forest Reserve (AHFR), Selangor

**FIGURE 6:**
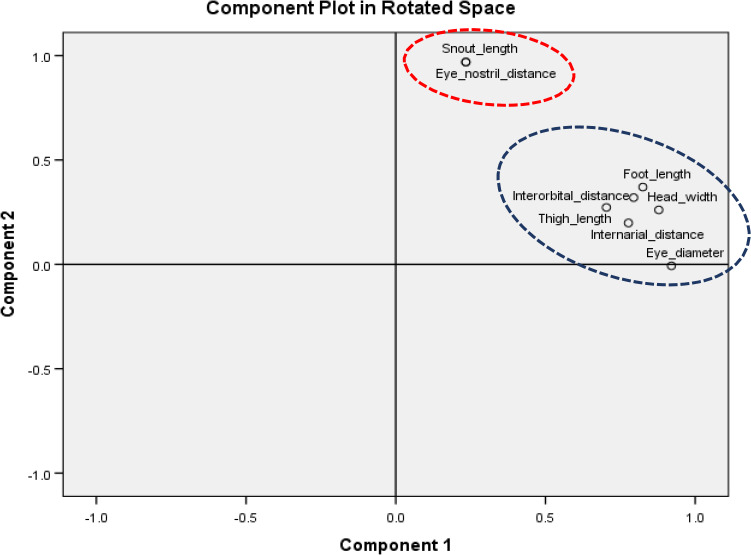
Component matrix of morphometric parameters in Pasoh Forest Reserve (PFR), Negeri Sembilan

PCA analysis of 15 morphometric traits for AHFR showed that the traits could be group into two components: Component 1 that comprised of head length, eye diameter, head width, internarial distance, interorbital distance, forearm length, tibia length, foot length, and thigh length; Component 2, which consist of the snout length and eye‐nostril distance (Table 7; Figure 5). Four morphometric traits, snout‐vent length, tympanum diameter, upper eyelid width, and hand length, were not included in any components due to the low value of loading factors (≥0.40 on more than one component). In Table 6; Figure 6, the component matrix was represented by two components for PFR. Component 1 comprised eye diameter, head width, internarial distance, interorbital distance, foot length, and thigh length. Component 2 included the snout length and eye‐nostril distance. Seven morphometric traits, snout‐vent length, head length, tympanum diameter, upper eyelid width, hand length, forearm length, and tibia length, were not included in any components due to low value of loading factors (≥ 0.40 on more than one component). All morphometric traits that grouped in the same component were positively correlated to each other (Figures 5 and 6; Table 8).

**TABLE 7:** Rotated Component matrix of morphometric parameters in Ayer Hitam Forest Reserve (AHFR), Selangor

Morphometric traits	Component	
	1	2
Head length	0.74	–
Snout length	–	0.91
Eye‐nostril distance	–	0.91
Eye diameter	0.77	–
Head width	0.92	–
Internarial distance	0.84	–
Interorbital distance	0.83	–
Forearm length	0.90	–
Tibia length	0.91	–
Foot length	0.80	–
Thigh length	0.70	–

Note

–, Not applicable.

**TABLE 8:** The independent sample *t* test related to morphometric traits and sex of *Kalophrynus palmatissimus* in Pasoh Forest Reserve (PFR), Negeri Sembilan (*n* of males = 20), (*n* of females = 11)

Variables	Sex	Mean (mm)	*SD*	*t*	*p*‐value
Head width	Male	0.66	0.11	−2.351	.026
	Female	0.76	0.13		
Snout‐vent length	Male	2.86	0.56	−2.351	.026
	Female	3.35	0.54		
Tibia length	Male	1.16	0.22	−1.871	.071
	Female	1.31	0.20		
Interorbital distance	Male	0.36	0.07	−1.698	.100
	Female	0.40	0.05		
Head length	Male	0.65	0.11	−2.187	.037
	Female	0.74	0.11		
Eye diameter	Male	0.26	0.06	−3.043	.005
	Female	0.33	0.06		
Internarial distance	Male	0.19	0.04	−2.728	.011
	Female	0.23	0.03		
Eye‐nostril distance	Male	0.25	0.06	0.358	.723
	Female	0.25	0.04		
Foot length	Male	0.92	0.18	−1.591	.122
	Female	1.03	0.21		
Tympanum diameter	Male	0.23	0.06	−1.148	.260
	Female	0.26	0.06		
Thigh length	Male	1.31	0.24	−2.230	.034
	Female	1.52	0.27		
Snout length	Male	0.35	0.06	0.358	.723
	Female	0.35	0.04		
Hand length	Male	0.66	0.10	−1.350	.187
	Female	0.71	0.11		
Forearm length	Male	0.78	0.18	−1.686	.103
	Female	0.89	0.16		
Upper eyelid width	Male	0.21	0.06	−1.370	.181
	Female	0.24	0.04		

## 4 DISCUSSION

The species of *K. palmatissimus* identified were based on several characteristics. Kiew (1984) described that *K. palmatissimus* are differed from *K. pleurostigma* by being smaller, have a more pointed snout, shorter head, smaller tympanum, shorter arm, and more webbing on the feet. It concluded that the most distinctive features are the degree of webbing on its feet and its more pointed snout. A total of 65 samples of *K. palmatissimus* from the two forest reserves were examined. *K. palmatissimus* recorded at AHFR were 20 males and 14 females, while those recorded at PFR were 20 males and 11 females.

*K. palmatissimus* in Ayer Hitam Forest Reserve, Puchong, Selangor (AHFR) were bigger as compared to the *K. palmatissimus* in Pasoh Forest Reserve, Negeri Sembilan (PFR).

This variation may be due to differences in climatic factors and the physical environment of the two forest reserves. AHFR's mean humidity and rainfall were higher than at PFR. Meanwhile, AHFR's mean temperature was lower than at PFR. The AHFR is surrounded by a construction area infrastructure, while PFR is surrounded by an oil palm plantation. Wang et al. (2014) suggested that factors that possibly influence the microclimates are the distribution of buildings and vegetation. It is known that morphometric differentiation of amphibians is correlated by climatic and ecological conditions (Amor et al., 2009) and a combination of genetic and environmental factors (Savage et al., 2015; Stock et al., 2008).

The 15 measured morphometric traits were positively correlated with each other. It suggested that as the frogs grow, they become bigger, and their morphometric traits will directly increase. Snout‐vent length (SVL) had significantly influenced other morphometric traits of *K. palmatissimus*, except for hand length. When the snout‐vent length of *K. palmatissimus* grows, the other morphometric traits, which are head length, snout length, eye‐nostril distance, eye diameter, tympanum diameter, head width, internarial distance, interorbital distance, upper eyelid width, forearm length, tibia length, foot length, and thigh length, will simultaneously grow together. This supported the correlation results and suggested that all morphometric traits grow simultaneously in *K. palmatissimus*. This phenomenon was noted previously by Vega‐Trejo et al. (2013), who indicated that the morphology of anurans change as they grow. Marshall et al. (2018) mentioned that the reduction in body size of anurans followed by a reduction of some morphological traits, such as skull elements and the number of digits.

The PCA results grouped the morphometric traits of *K. palmatissimus* into two components. Component 1 of AHFR, the increasing size of head width influenced the size of all morphometric traits of *K. palmatissimus*. This result seemed to be similar in Component 2 as increasing size of snout length influenced the size of eye‐nostril distance. In Component 1 of PFR, the increasing size of eye diameter influenced the size of other morphometric traits of *K. palmatissimus*, while the increasing size of snout length influenced the size of eye‐nostril distance in Component 2. The results identified that head width and eye diameter as the traits that influenced most other morphometric traits of *K. palmatissimus* at both forest reserves. Zug and Duellman (2018) defined that anurans' broad head had influenced their movement as long and powerful foot of anurans will push the fused head and trunk in a forward trajectory. During feeding, anurans pull their eyes down into the roof of their mouth to help to shove the food down their throat (Zug & Duellman, 2018). Hence, the eyes of most species are large and well developed along with their foot and thigh lengths.

The Spearman correlation test showed that only the temperature negatively associated (*p* < .05) with all 15 morphometric traits. Meanwhile, soil pH had positive associations (*p* < .05) with a few morphometric traits of *K. palmatissimus*. Recent evidence suggested that for ectotherms, such as frogs, the low temperatures result in larger cells, which may increase their body size (Hessen et al., 2013). In turn, body size affects survival, locomotion, and reproductive success (Wikelski & Romero, 2003). Therefore, the temperature can be an important selective factor, and testing on how physiological traits, morphology, and locomotion reaction to different thermal environments, can provide a better understanding of local adaptations of anurans (Angilletta et al., 2004). Anderson and Johnson (2018) argued that soil pH was found to be important as an abiotic factor that affects the growth and survival of Marbled Salamanders. As soil acidity increased, the salamander body development and survival will decline.

At AHFR, male and female *K. palmatissimus* demonstrated statistically significant differences for only head length (HL), eye diameter (ED), and head width (HW). The statistical results were the same for PFR, except for snout‐vent length (SVL), internarial distance (IND) and thigh length (THL) also showed a significant difference. The study proposed that there was sexual dimorphism between males and females *K. palmatissimus* at AHFR and PFR. Sexual dimorphism in anuran is where the male and female differ in body size and this difference is attributed to sexual selection (Bell & Zamudio, 2012).

However, a limitation in this study is that the species of *K. palmatissimus* is restricted to only undisturbed areas. For example, the 50‐hectare plot, which is one of the study areas in Compartment 32 at PFR, has relatively high stem density, where large numbers of trees have been logged over the recent years. Manokaran and LaFrankie (1990) mentioned that the disturbance rates increased in Compartment 32 of PFR in recent years. The logging activities throughout the years may have caused a decrease in the number of anurans, especially *K. palmatissimus*. The sample size of *K. palmatissimus* recorded from the two forest reserves were limited. During the previous study in Korea, Borzee et al. (2013) reported that the total number of endangered males *H. japonica* and *H*. *suweonensis* were 41 and 47, respectively, and are representative of the entire distribution of *H*. *suweonensis*.

## 5 CONCLUSIONS

The present study showed the comparison of 15 morphometric traits of *K*. *palmatisssimus* at two forest reserves (AHFR and PFR), with *K. palmatissimus* at AHFR were slightly larger than at PFR. Furthermore, the females and males of *K. palmatissimus* were found to be sexually dimorphic. Still, all morphometric traits had simultaneous growth, and the temperature had a negative influence on all morphometric traits, whereas soil pH had a positive effect on a few morphometric traits. It suggested that the information from this study could contribute to a better understanding of *K. palmatissimus* morphologies characteristics and the influence of climatic factors (soil pH and temperature) on morphological characteristics of *K. palmatissimus* at the two forest reserves. This could help future conservation programs and management to protect this endemic species from extinction.

## CONFLICT OF INTEREST

None declared.

## AUTHOR CONTRIBUTIONS

**Muhammad Faris Abdul Aziz:** Formal analysis (lead); investigation (lead); methodology (equal); resources (equal); software (lead); writing‐original draft (lead). **Marina Mohd. Top @ Mohd. Tah:** Data curation (equal); Funding acquisition (lead); project administration (lead); supervision (lead); visualization (equal); writing‐review & editing (equal). **Shamarina Shohaimi:** Funding acquisition (equal); methodology (equal); project administration (equal); software (equal); supervision (equal); writing‐review & editing (equal). **Nurul Izza Ab Ghani:** Formal analysis (equal); funding acquisition (equal); methodology (equal); project administration (equal); software (equal); supervision (equal); writing‐review & editing (equal). **Christine Fletcher:** Formal analysis (equal); funding acquisition (equal); methodology (equal); project administration (equal); supervision (equal); writing‐review & editing (equal).

## ETHICAL APPROVAL

This research approved by the Institutional Animal Care and Use Committee (IACUC) (Reference no: UPM/IACUC/AUP‐R007/2018) of Universiti Putra Malaysia and the Department of Wildlife and National Parks Peninsular Malaysia (PERHILITAN) (Permit no: HQ‐00131‐15‐70) ethical license.

## Data Availability

*Kalophrynus palmatissimus* locations and climatic data: https://doi.org/10.5061/dryad.4j0zpc8bj.
